# Physiological and Metabolic Responses of Alfalfa to Cold Stress Under Saline–Alkaline Conditions

**DOI:** 10.3390/ijms27010267

**Published:** 2025-12-26

**Authors:** Xu Zhuang, Dongmei Zhang, Ying Yang, Weibo Han, Linlin Mu, Zhongbao Shen, Guili Di, Yaling Liu, Jia You, Jianli Wang

**Affiliations:** 1 Institute of Forage and Grassland Sciences, Heilongjiang Academy of Agricultural Sciences, Harbin 150086, China; yangyangcaocao@haas.cn (X.Z.); zhangdongmei@haas.cn (D.Z.); hanweibo@haas.cn (W.H.); budaoweng@haas.cn (L.M.); shenzhongbao@haas.cn (Z.S.); 2 College of Life Sciences and Technology, Harbin Normal University, Harbin 150025, China; yangying1212@126.com; 3 Institute of Economic Crops, Heilongjiang Academy of Agricultural Sciences, Harbin 150086, China; diguili@haas.cn; 4 National Center of Pratacultural Technology Innovation (Under Preparation), Hohhot 010030, China; liuyaling0719@163.com

**Keywords:** alfalfa, saline-alkaline-cold stress, physiological characteristics, chlorophyll fluorescence, nitroblue tetrazolium staining, metabolites

## Abstract

Alfalfa (*Medicago sativa* L.), a perennial leguminous herb, can tolerate cold and saline–alkaline conditions. In this study, alfalfa cultivars LJ and 218TR were exposed to saline–alkaline, cold, and saline–alkaline–cold conditions and compared in terms of phenotypes, physiological indices, key metabolite contents, and stress-responsive gene expression. Malondialdehyde, soluble sugar, proline contents and phenylalanine ammonia-lyase (PAL), superoxide dismutase, catalase, and peroxidase activities initially increased under individual stress conditions, but decreased when stresses were combined. Photosystem II maximum photochemical efficiency and chlorophyll contents decreased under individual and combined stress conditions. Nitroblue tetrazolium-stained leaves revealed that the combined stress treatment significantly increased cell mortality rates and superoxide anion levels. LJ was more tolerant to saline–alkaline, cold, and combined stress treatments than 218TR. Metabolite analyses indicated that for LJ and 218TR, salicylic acid (SA) was the most responsive metabolite to combined stress conditions. Additionally, the expression of *isochorismate synthase* (*ICS*) and *PAL* genes critical for SA biosynthesis was upregulated under single or combined stress conditions, leading to SA accumulation and improved tolerance to saline–alkaline–cold conditions. This study revealed the physiological indices and molecular changes underlying alfalfa responses to saline–alkaline stress combined with cold stress, providing a theoretical basis for breeding stress-tolerant cultivars.

## 1. Introduction

Soil salinization–alkalinization, which is characterized by the accumulation of soluble salts from subsoil and groundwater layers in the surface layer, is a serious global ecological and agricultural issue that significantly inhibits plant growth and decreases crop yield and quality [1]. Saline–alkaline soils may adversely affect plant organ development, dry matter accumulation, and critical physiological processes [2], which can lead to damaged antioxidant defense systems; suppressed photosynthesis; imbalanced mineral/ion absorption, use, distribution, and translocation in roots [3]; and relatively inefficient nutrient uptake [4]. In saline–alkaline environments, a high soil pH exacerbates osmotic stress, leading to soil compaction as well as plant dehydration, ionic imbalance, and restricted root growth and nutrient absorption, ultimately causing leaf wilting and plant death [5,6].

Northeast China, particularly the Songnen Plain, is one of the main regions with saline–alkaline soil, in which Na_2_CO_3_, NaHCO_3_, Na_2_SO_4_, and NaCl are the most abundant salts [7,8] and pH levels range from 8.5 to 10.5 [9]. Additionally, relatively long and cold winters in this region result in low overwintering rates, unstable plant survival rates, and frequent damages due to cold/freezing conditions. The harmful effects of cold stress on plants include membrane damage, metabolic dysregulation, water imbalance, oxidative stress, and growth inhibition. Low temperatures can decrease membrane fluidity, increase permeability, suppress photosynthesis and respiration, disrupt the water balance, and trigger reactive oxygen species (ROS) accumulation, ultimately impairing plant growth and development. To protect against cold stress, plants have complex physiological and molecular mechanisms that induce various processes, including calcium signaling, hormone regulation, cold-responsive gene expression, osmolyte accumulation, and synthesis of protective proteins, which collectively mitigate damage and maintain cellular stability [10].

Alfalfa, an important leguminous forage crop [11], is renowned for its high yield, strong regrowth, long lifespan, and palatability, playing a key role in China’s livestock industry. Although alfalfa is moderately tolerant to saline–alkaline conditions and contributes to soil remediation, the combined effects of saline–alkaline and cold stresses make cultivating alfalfa in cold, high-latitude northern regions challenging. Severe cold stress inhibits growth, disrupts metabolism, and can lead to plant death. Therefore, breeding alfalfa cultivars tolerant to both saline–alkaline and cold stresses is critical for enhancing alfalfa production in northern China.

In a previous study, two alfalfa cultivars that differ in terms of their tolerance to saline–alkaline conditions were analyzed. On the basis of physiological indices, LJ and 218TR were revealed to be the most and least tolerant cultivars among 15 analyzed cultivars. In the current study, the effects of saline–alkaline soil treatments on these two cultivars under cold conditions were compared by analyzing phenotypic and physiological characteristics. Additionally, key metabolic pathways and genes responsive to saline–alkaline–cold conditions were identified via metabolomics and RT-qPCR analyses. The resulting data clarified the molecular mechanism through which alfalfa plants adapt to the simultaneous exposure to abiotic stresses. We hypothesized that the combined effects of saline–alkaline and cold stresses on alfalfa differ from the effects of a single stress. The study findings provide theoretical and practical insights relevant to breeding alfalfa germplasm resistant to saline–alkaline–cold conditions and advancing molecular breeding strategies, thereby supporting the sustainable development of the alfalfa industry in challenging environments.

## 2. Results

### 2.1. Alfalfa Plant Phenotypes Under Saline–Alkaline and Cold Stress Conditions

**Figure 1:**
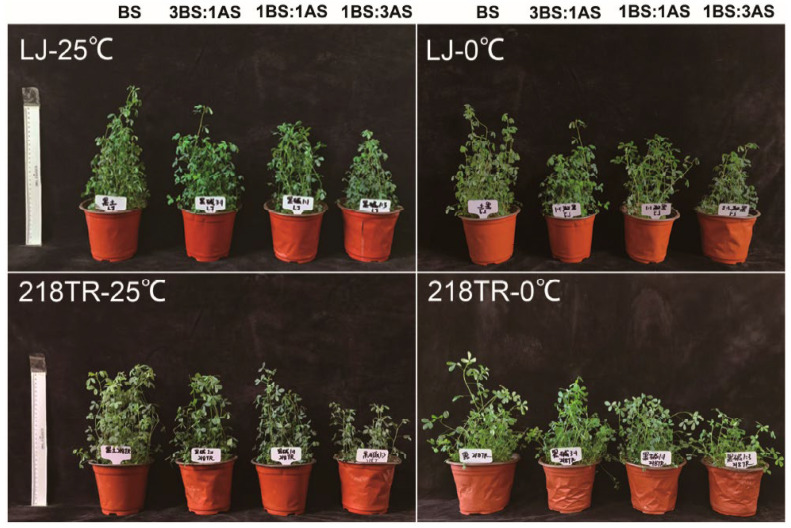
Phenotypes of alfalfa cultivars LJ and 218TR exposed to different saline–alkaline and temperature conditions. BS for black soil, AS for alkaline soil.

Both LJ and 218TR grew in saline–alkaline soils at 25 °C (Figure 1). However, LJ and 218TR growth was gradually inhibited as the severity of saline–alkaline stress conditions increased. Notably, saline–alkaline stress inhibited the growth of 218TR more than the growth of LJ. After exposure to 0 °C (low-temperature stress), no significant leaf wilting was observed for LJ and 218TR under different saline–alkaline conditions. Slight lodging was detected for 218TR, but not for LJ (Figure 1). This suggests that LJ was more tolerant to saline–alkaline conditions combined with low-temperature stress.

### 2.2. Physiological Characteristics of Alfalfa Plants Exposed to Saline–Alkaline and Cold Stresses

**Figure 2:**
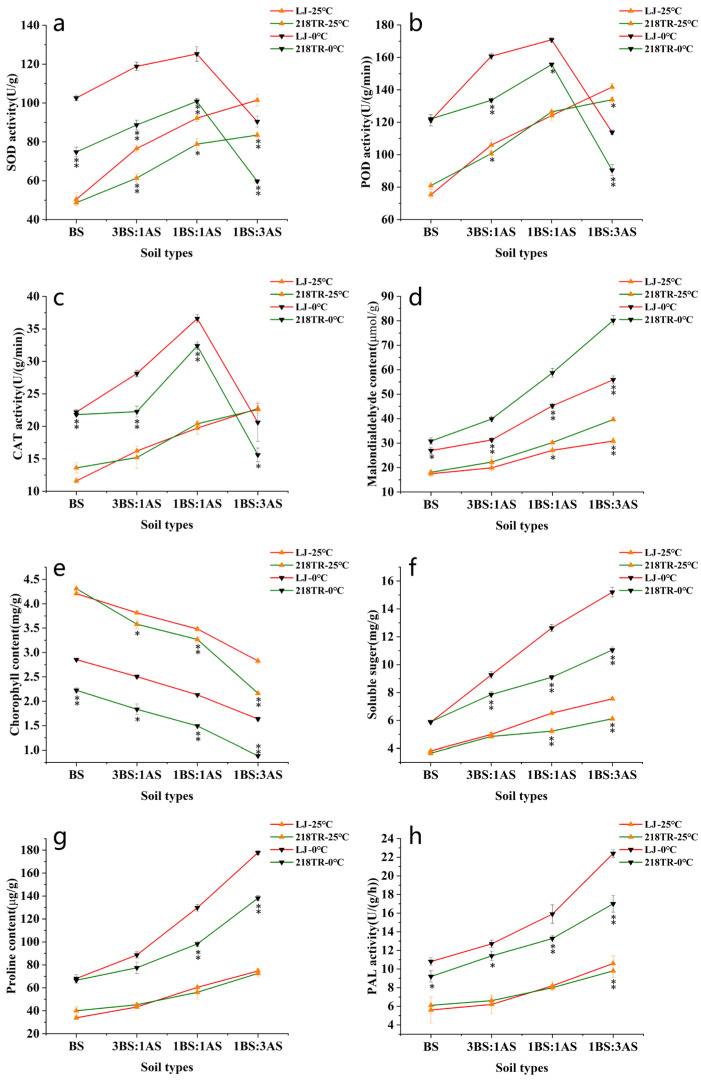
Physiological indices of alfalfa cultivars LJ (pink line) and 218TR (green line) exposed to different saline–alkaline and cold stresses. The 25 °C treatments for both cultivars are represented by upright arrows, and the 0 °C treatments by inverted arrows. Leaf tissues were used to analyze SOD, POD, CAT, and PAL activities as well as MDA, chlorophyll, soluble sugar, and proline contents. BS, black soil; AS, alkaline soil. (**a**): SOD activity, (**b**): POD activity, (**c**): CAT activity, (**d**): MDA content, (**e**): chlorophyll content, (**f**): soluble sugar content, (**g**): proline content, (**h**): PAL activity. Data are presented as the mean ± SE from three replicates. Asterisks indicate significant differences as determined by Student’s *t*-test (* 0.01 < *p* < 0.05, ** *p* < 0.01).

Superoxide dismutase (SOD), peroxidase (POD), catalase (CAT), and phenylalanine ammonia-lyase (PAL) activities as well as malondialdehyde (MDA), soluble sugar (SS), and proline (Pro) contents of both alfalfa cultivars tended to increase as the severity of the saline–alkaline stress increased, peaking under 1BS:3AS conditions. For both cultivars, the chlorophyll (Chl) content tended to decrease as the severity of the saline–alkaline stress treatment increased, reaching its lowest level under 1BS:3AS conditions at 25 °C. After exposure to 0 °C, the SOD, POD, and CAT activities of both cultivars gradually increased under BS, 3BS:1AS, and 1BS:1AS conditions, but decreased in response to 1BS:3AS. The increases in SOD, POD, and CAT activities were greater for LJ than for 218TR under saline–alkaline conditions combined with cold stress (Figure 2a–c, *p* < 0.05). In addition, MDA, Pro, and SS contents and PAL activity increased in both alfalfa cultivars. Pro and SS contents and PAL activity were higher in LJ than in 218TR, but the increase in the MDA content was greater in 218TR than in LJ under saline–alkaline conditions combined with cold stress (Figure 2d,f–h, *p* < 0.05).

LJ and 218TR were subsequently subjected to low-temperature stress (0 °C), which resulted in a decrease in the Chl content of both cultivars. A comparison of the Chl content under different soil conditions at 25 °C and after exposure to 0 °C indicated that decreases in Chl contents under BS, 3BS:1AS, 1BS:1AS, and 1AS:3BS conditions were greater for LJ than for 218TR (Figure 2e, *p* < 0.05).

### 2.3. Chlorophyll Fluorescence of Alfalfa Under Saline–Alkaline and Cold Stress Conditions

**Figure 3:**
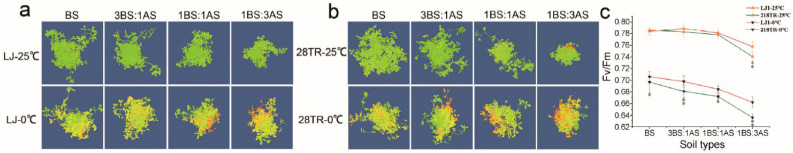
Chlorophyll fluorescence of alfalfa cultivars LJ and 218TR under different saline–alkaline and cold stress conditions. The upper row of (**a**,**b**) shows the chlorophyll fluorescence image of LJ and 218TR under saline–alkali stresses (25 °C). The lower row of (**a**,**b**) shows the chlorophyll fluorescence image of LJ and 218TR under combined saline–alkali and cold stresses (0 °C). (**c**) shows the Fv/Fm value of LJ and 218TR under saline–alkali stresses and combined stress. Asterisks indicate significant differences as determined by Student’s *t*-test (* 0.01 < *p* < 0.05, ** *p* < 0.01).

The photosystem II complexes (PSII) and Fv/Fm values of LJ and 218TR were similar under BS, 1BS:3AS, and 1BS:1AS conditions (Figure 3), but the PSII of 218TR was more damaged than that of LJ under 1BS:3AS conditions (Figure 3a,b). In addition, the Fv/Fm value of 218TR was significantly lower than that of LJ following exposure to the 1BS:3AS condition (Figure 3c, *p* < 0.05). Additionally, the photosynthesis of LJ was significantly less inhibited than 218TR under combined saline–alkaline conditions with cold stress (Figure 3, *p* < 0.05).

### 2.4. Nitroblue Tetrazolium (NBT) Staining of Alfalfa Under Saline–Alkaline and Cold Stress Conditions

**Figure 4:**
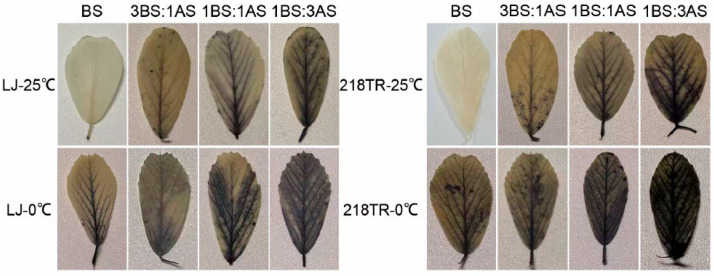
NBT staining of alfalfa cultivars LJ and 218TR under different saline–alkaline and temperature stress conditions.

The NBT staining method is based on the reaction between NBT and intracellular O_2_^−^, which is detected as a blue stain [12]. To assess the extent of cellular damage in alfalfa under saline–alkaline, cold, and saline–alkaline–cold stress conditions, the leaves of alfalfa cultivars LJ and 218TR were stained with NBT to measure the accumulation of O_2_^−^ in leaf cells. At 25 °C, O_2_^−^ accumulation increased in both LJ and 218TR as the severity of saline–alkaline conditions increased, peaking after the 1BS:3AS treatment. Following an exposure to 0 °C, the accumulation of O_2_^−^ in LJ and 218TR increased significantly (compared with the accumulation after an exposure to saline–alkaline stress alone). A comparison of O_2_^−^ levels under different saline–alkaline conditions at 25 and 0 °C indicated that the rank order of O_2_^−^ accumulation in both LJ and 218TR was as follows (highest to lowest): 1BS:3AS > 1BS:1AS > 3BS:1AS > BS. Additionally, the O_2_^−^ level was higher in 218TR than in LJ under all conditions (Figure 4).

### 2.5. Analysis of Alfalfa Metabolites Under Saline–Alkaline and Cold Stress Conditions

The upregulation and downregulation of 49 metabolites in both LJ and 218TR were statistically analyzed. The comparison between the LJ-BS-25 °C group and the LJ-1BS:3AS-25 °C group detected 18 upregulated metabolites and 31 downregulated metabolites (Table 1). By contrast, the comparison between the LJ-BS-25 °C group and the LJ-BS-0 °C group revealed 27 upregulated metabolites and 22 downregulated metabolites. According to the comparison between the LJ-1BS:3AS-25 °C group and the LJ-1BS:3AS-0 °C group, 30 metabolites were upregulated and 19 metabolites were downregulated. In the comparison between the LJ-BS-0 °C group and the LJ-1BS:3AS-0 °C group, 33 metabolites were upregulated, whereas 16 metabolites were downregulated.

The comparison between the 218TR-BS-25 °C group and the 218TR-1BS:3AS-25 °C group identified 14 metabolites that were upregulated as well as 35 metabolites that were downregulated. The comparison between the 218TR-BS-25 °C group and the 218TR-BS-0 °C group detected 20 upregulated metabolites and 29 downregulated metabolites. In the comparison between the 218TR-1BS:3AS-25 °C group and the 218TR-1BS:3AS-0 °C group, 36 metabolites were upregulated, while 13 metabolites were downregulated. The comparison between the 218TR-BS-0 °C group and the 218TR-1BS:3AS-0 °C group detected 34 upregulated metabolites and 15 downregulated metabolites. These results imply that metabolite contents tended to increase under saline–alkaline–cold stress conditions, and were used by plants to defend against stress. Among all eight treatment groups, there were three upregulated hormones that appeared consistently, namely GA4, IAM, and SA.

### 2.6. Heatmap of Metabolite Contents Under Saline–Alkaline and Cold Stress Conditions

**Figure 5:**
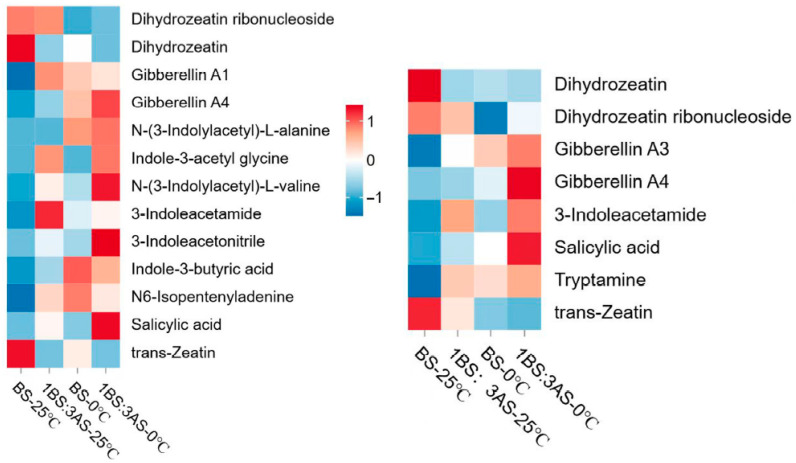
Heatmap of metabolite contents in LJ (**left**) and 218TR (**right**) under different saline–alkaline and temperature stress conditions.

Among the analyzed metabolites in LJ, the contents of gibberellin GA4, N-(3-indolylacetyl)-L-alanine, indole-3-acetyl glycine, N-(3-indolylacetyl)-L-valine, 3-indoleacetonitrile, and salicylic acid (SA) increased under saline–alkaline–cold stress conditions (Figure 5, left). Of the examined metabolites in 218TR, the contents of gibberellins GA3 and GA4, 3-indoleacetamide, and SA changed significantly in response to the exposure to saline–alkaline–cold stress conditions, with GA4 and SA contents increasing significantly (Figure 5, right). The relatively high SA contents in both LJ and 218TR may reflect the importance of this phytohormone for protecting alfalfa from saline–alkaline–cold stress.

### 2.7. Expression of Key Genes in the SA Biosynthesis Pathway

**Figure 6:**
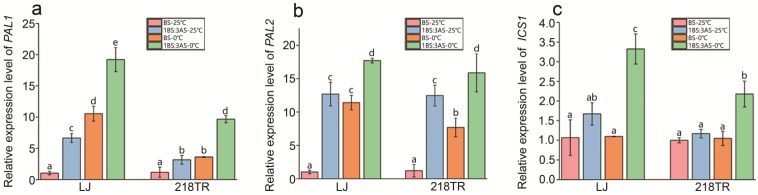
Relative *PAL1*, *PAL2*, and *ICS1* expression levels in LJ and 218TR under different saline–alkaline and cold stress conditions. (**a**): Relative expression level of *PAL1*; (**b**): Relative expression level of *PAL2*; (**c**): Relative expression level of *ICS1*. Data are presented as the mean ± SE of three independent replicates. A one-way ANOVA was performed to analyze data (Duncan’s post hoc HSD test), with significant differences (*p* < 0.05) indicated by lowercase letters.

The isochorismate synthase (ICS) pathway and the PAL pathway are two critical routes for SA biosynthesis. In this study, the expression levels of *ICS1*, which is a key gene in the ICS pathway, as well as *PAL1* and *PAL2*, which are important genes in the PAL pathway, in LJ and 218TR under different treatment conditions were analyzed. According to RT-qPCR data, *PAL1* and *PAL2* expression levels in LJ and 218TR increased significantly in response to saline–alkaline stress, low-temperature stress, and saline–alkaline stress combined with low-temperature stress (relative to the corresponding expression in the control group) (Figure 6a,b). Similarly, *ICS1* expression levels in LJ and 218TR increased significantly under saline–alkaline–cold stress conditions (Figure 6c).

## 3. Discussion

### 3.1. Physiological Responses of Alfalfa to Saline–Alkaline and Cold Stress Conditions

Photosynthesis is an essential physiological process in plants, with Chl serving as the foundation of photosynthesis and a marker of the plant photosynthetic capacity [13]. Under normal growth conditions, Chl contents are maintained in a dynamic equilibrium, but environmental stress can disrupt this balance, leading to changes in Chl contents [14]. These changes can indicate the effects of stress factors on plants, thereby reflecting plant stress tolerance [15]. Previous studies showed that the Chl content of alfalfa varieties decreases significantly following an exposure to saline–alkaline conditions [16,17]. The current study also detected significant decreases in the Chl content as the severity of saline–alkaline stress and cold stress conditions increased. The decrease in Chl contents was greater for 218TR than for LJ under saline–alkaline and cold conditions.

Chl fluorescence reflects the relationship between plant photosynthesis and environmental conditions [18,19] as well as photosynthesis-related physiological characteristics of plants [20]. Saline–alkaline stress decreases the Fv/Fm value, thereby affecting PSII efficiency; this decrease is a useful indicator of the severity of saline–alkaline stress [21]. In an earlier study, the Fv/Fm value decreased for five *Bromus inermis* seedlings treated with salt stress [22]. A simultaneous exposure to salinity and low temperatures can severely disrupt melon seedling growth and decrease Fv/Fm values [23]. In the present study, the Fv/Fm values of both alfalfa cultivars decreased as the severity of saline–alkaline conditions increased at 25 °C; however, the difference in Fv/Fm values before and after the exposure to stress was small, implying that alfalfa plants can effectively maintain PSII activities and the potential maximum photochemical efficiency under certain saline–alkaline stress levels, ultimately stabilizing photosynthetic reactions to prevent a significant decline in the net photosynthetic rate [24]. However, when saline–alkaline stress conditions increase to a specific threshold, the Fv/Fm value decreases rapidly. Similarly, at 0 °C, the Fv/Fm value is substantially affected by saline–alkaline stress, with a significant decrease as the severity of the stress conditions increases (the range of Fv/Fm is 0.60–0.69). In this study, LJ was more tolerant to saline–alkaline–cold stress conditions than 218TR. In addition, the PSII complex of alfalfa was unaffected unless saline–alkaline conditions were severe. Moreover, PSII photoinhibition increased when saline–alkaline stress was combined with cold stress.

Stress conditions can lead to excessive ROS production in plants, resulting in the accumulation of free radicals, enhanced membrane lipid peroxidation, and inactivation or degradation of critical enzymes and proteins, with detrimental effects on plant growth and development [24,25,26]. Plants possess membrane-protecting enzymes, including SOD, POD, and CAT, that scavenge ROS [27]. These enzymes convert excess ROS into harmless substances, such as H_2_O, which helps to maintain ROS metabolism and protect plants from ROS-induced damage. Changes in antioxidant enzyme activities reflect the extent of oxidative damage in plants, while also serving as an important indicator of abiotic stress responses [28]. A previous study on peanut seedlings showed that after treatments with high salinity or drought stress, SOD, CAT, and POD activities initially increased, but then decreased [29]. These findings are consistent with data generated in the current study. Thus, increasing the severity of saline–alkaline conditions can adversely affect the enzyme system, exacerbate membrane lipid peroxidation, and increase membrane damage [30]. Notably, genotypes tolerant to saline–alkaline stress (e.g., LJ) maintain relatively high antioxidant enzyme activities under stress conditions, thereby mitigating damage due to membrane lipid peroxidation. Ascorbate peroxidase and glutathione reductase are also important antioxidant enzymes that scavenge ROS. Although we did not analyze their activities, these enzymes are functionally similar to SOD, CAT, and POD. Therefore, ascorbate peroxidase and glutathione reductase activities may be indirectly determined by analyzing SOD, CAT, and POD activities.

Our comprehensive analysis of various indicators revealed an interaction between saline–alkaline stress and cold stress. Saline–alkaline stress can enhance the inhibitory effect of cold stress on alfalfa. Thus, combining saline–alkaline stress with cold stress can further suppress alfalfa growth and development through additive effects. Under stress conditions, SS and Pro contents as well as PAL, SOD, POD, and CAT activities increased more in LJ than in 218TR. By contrast, the increase in the MDA content was lower in LJ than in 218TR. These results indicate that LJ can tolerate saline–alkaline, cold, and saline–alkaline–cold stresses better than 218TR.

The accumulation of ROS (O_2_^−^) in leaves under stress conditions can indicate the extent of leaf damage. NBT staining is useful for localizing ROS within tissues [31]. In the current study, LJ and 218TR leaves were stained with NBT to examine cell death and O_2_^−^ accumulation in cells in response to single or combined stress conditions.

Osmotic regulation is one of the most fundamental characteristics related to plant stress tolerance; enhanced osmotic regulation is an important mechanism for improving plant stress resistance [32]. The most common osmotic regulators include Pro, SS, and PAL. Previous research showed that in ryegrass seedlings, SS and Pro contents as well as PAL activity increase under freeze–thaw or NaHCO_3_ stress conditions, but these increases are greater when these two stresses are combined [33]. In addition, in creeping bentgrass, Pro and MDA contents are higher after an exposure to both high-temperature and drought stresses than after an exposure to only one of these stresses [34]. In this study, SS and Pro contents as well as PAL activity increased after stress treatments, while increases in MDA, SS, and Pro contents and PAL activity were significantly higher under combined stress treatment conditions than under saline–alkaline or low-temperature stress conditions alone [33,34].

### 3.2. Metabolic Responses of Alfalfa to Saline–Alkaline and Cold Stresses

Numerous studies have demonstrated that endogenous hormones play a crucial role in plant responses to stress conditions. For example, in pea roots exposed to stress, endogenous hormone contents decrease, but ABA and IAA levels increase under drought conditions [35]. SA also plays a key role in plant responses to abiotic stressors, including high and low temperatures, salinity–alkalinity, and heavy metals. Additionally, in *Arabidopsis*, endogenous SA rapidly accumulates following an exposure to low-temperature stress [36]. In this study, IAA-Ala, IAA-Gly, IAA-Val, gibberellin GA4, and 3-indoleacetamide contents in LJ as well as gibberellin GA3, GA4, and 3-indoleacetamide contents changed significantly in response to saline–alkaline, cold, and combined stress treatments. Moreover, SA contents were relatively high in both LJ and 218TR, indicating that hormones, including SA, were the most relevant metabolites involved in alfalfa responses to saline–alkaline, cold, and combined stress treatments.

The ICS and PAL pathways are two important routes for SA biosynthesis in plants. Earlier studies indicated that in *Arabidopsis*, the ICS pathway is the most common route for SA synthesis in response to biotic and abiotic stresses [37,38]. By contrast, in soybean, these two pathways contribute equally to SA biosynthesis [39]. However, the PAL pathway is the primary route for SA synthesis in tobacco according to a previous study that investigated *ICS* and *PAL* expression [40]. On the basis of the data generated in the present study, *PAL1* and *PAL2* expression levels were upregulated by saline–alkaline, cold, and combined stress treatments, while *ICS1* expression increased after the combined stress treatment (relative to the corresponding expression in the control). Upregulated *PAL1* and *PAL2* expression leads to increased PAL activity, which causes SA to accumulate. Therefore, increases in PAL activity can improve the tolerance of alfalfa plants to saline–alkaline and cold stress conditions. These results suggest that PAL and ICS pathways may be crucial for alfalfa tolerance to saline–alkaline and cold conditions because they contribute to SA biosynthesis and accumulation.

## 4. Materials and Methods

### 4.1. Materials and Treatments

Saline–alkaline stress-tolerant and -sensitive alfalfa cultivars (LJ and 218TR, respectively) were provided by the Forage Breeding Laboratory, Institute of Forage and Grassland Sciences, Heilongjiang Academy of Agricultural Sciences. These two cultivars were selected after screening 15 alfalfa germplasm resources on the basis of their responses to saline–alkaline conditions. According to pH values [41], saline–alkaline conditions were classified as mild (pH 7.5–8.5), moderate (pH 8.5–9.5), and severe (pH > 9.5). To simulate these stress levels, black soil (BS) and alkaline soil (AS) were mixed at mass ratios of 3:1 (mild), 1:1 (moderate), and 1:3 (severe), with BS alone serving as the control. 218TR and LJ seeds were sown in pots (11 cm diameter × 11 cm depth; soil volume of 1045 cm^3^) containing the four soil types. Each stress treatment comprised six replicates for a total of 48 samples. Plants were cultivated under the following conditions: 16 h light (light intensity, 15,000 lx; 25 °C)/8 h dark (22 °C) photoperiod, with regular watering.

During the 3–4 true leaf stage, three replicates per treatment were acclimated to cold conditions, while the remaining three replicates were maintained under ambient conditions. More specifically, to generate cold-acclimated plants, samples were subjected to stepwise temperature decreases in a climate chamber (15,000 lx, 16 h light/8 h dark cycle) initially set at 25 °C. The temperature was decreased in 5 °C increments (i.e., 20, 15, 10, and 5 °C) at a rate of 1 °C/h, with each temperature held for 24 h to prevent thermal shock. Cold-acclimated plants were then exposed to cold stress (0 °C) for 24 h. Leaves were collected from all plants and then stored at −80 °C until analyzed.

### 4.2. Analyses of Phenotypes, Physiological Indices, and Metabolite Contents

After the same saline–alkaline and cold stress treatments, LJ and 218TR plants were photographed and their phenotypes were compared. In addition, leaf samples were collected from both cultivars for an analysis of physiological indices. SOD activity was examined according to the inhibition of NBT photochemical reduction [42], whereas POD activity was measured via guaiacol oxidation monitoring at 470 nm [43]. CAT levels were determined through the ultraviolet spectrophotometric tracking of H_2_O_2_ decomposition at 240 nm [44]. Lipid peroxidation was evaluated by measuring the MDA content according to TBA reactive substance formation, while free proline accumulation was assessed via sulfosalicylic acid extraction coupled with ninhydrin staining [45,46]. Photosynthetic pigments were extracted using an ethanol immersion method and then quantified spectrophotometrically at specific wavelengths (663, 645, and 470 nm) [47]. SS contents were determined by performing an anthrone–sulfuric acid colorimetric assay [48]. Pro contents were determined using a published acidic ninhydrin-based method [49]. PAL activity was measured using an ultraviolet spectrophotometer (Puxi, Beijing, China). ROS distribution was visualized through histochemical staining, with NBT used to detect superoxide radicals (O_2_^−^) to assess membrane integrity [50].

Chlorophyll fluorescence parameters (Fv and Fm) were examined using a Plant Explorer Pro high-throughput scanning system (PhenoTrait, Beijing, China). Photosynthetic efficiency (Fv/Fm) was measured under saline–alkaline and cold stress conditions. Before scanning, plants were adapted to darkness for 20–30 min. Samples were placed at the center of the imaging area and then the “Chlorophyll Fluorescence Measurement Mode” was selected. The recommended excitation light intensity was used (3000–4000 μmol m^−2^ s^−1^ at 25 cm). The instrument automatically collected Fo (initial fluorescence) and Fm (maximum fluorescence) values and calculated Fv/Fm values (Fv = Fm − Fo). Finally, data were exported and then mean Fv/Fm values along with fluorescence images of target areas were extracted.

Qualitative and quantitative analyses of metabolites were performed using an ultra-high-performance liquid chromatography–triple quadrupole tandem mass spectrometry system (Agilent 1290–6470 UHPLC-MS/MS, Santa Clara, CA, USA). For the chromatographic separation, a ZORBAX Eclipse Plus C18 column (2.1 × 100 mm, 1.8 μm; Agilent Technologies, Santa Clara, CA, USA) was maintained at 40 °C. Mobile phase A consisted of 0.05% formic acid in water, whereas mobile phase B was methanol. The gradient elution program was as follows: 0–2 min, 5% B; 2–8 min, 5–95% B; 8–10 min, 95% B; 10–10.1 min, 95–5% B; 10.1–12 min, 5% B, with a flow rate of 0.3 mL/min. Mass spectrometric detection was conducted using an electrospray ionization source in positive/negative modes with multiple reaction monitoring. Source parameters were as follows: capillary voltage, ±3500 V; nozzle voltage, 500 V; nebulizer gas temperature, 300 °C; nebulizer gas flow, 5 L/min; sheath gas temperature, 350 °C; and sheath gas flow, 11 L/min. Metabolite contents were determined according to standard curves constructed using gradient dilutions of the corresponding reference standards [51].

For molecular analyses, total RNA was isolated using TRIzol reagent (Invitrogen, Carlsbad, CA, USA), after which residual genomic DNA was removed and cDNA was synthesized using a TransScript One-Step SuperMix Kit (Transgen, Beijing, China). Gene-specific primers designed using Primer 5.0 were used for RT-qPCR analyses, which were completed on a Roche LightCycler 96 system (Roche, Basel, Switzerland). RT-qPCR reaction mixtures (20 μL) consisted of 10 μL 2× TransStart^®^ Tip Green qPCR SuperMix (TransGen, Beijing, China), 0.8 μL forward and reverse primers (10 μM), 2 μL cDNA template, and 6.4 μL nuclease-free water. The PCR program was as follows: initial denaturation at 95 °C for 30 s; 40 cycles of denaturation at 95 °C for 5 s and annealing/extension at 60 °C for 30 s (with fluorescence signal acquisition at the end of this step); melting curve analysis from 65 to 95 °C, with the signal acquired every 0.5 °C to confirm the specificity of amplified products. Relative target gene expression levels were calculated using the 2^−ΔΔCT^ method [52].

### 4.3. Data Processing and Analysis

Experimental data were processed using Excel 2020. Results are presented herein as the mean ± standard error. SPSS 27.0 software was used for an analysis of variance (ANOVA), with significance assessed at the 95% confidence level according to a one-way ANOVA. Duncan’s post hoc test was completed to evaluate the significance of differences among treatments in multiple comparisons (*p* < 0.05). Origin 2022 software was used for the graphical representation of data. Metabolite data were acquired and processed using Agilent MassHunter Workstation software (version B.10.0). Relative metabolite contents were log_2_-transformed and then visualized in heatmaps using the R version 4.5.2 package pheatmap. Differentially abundant metabolites were screened on the basis of the following criteria: fold-change ≥ 2 or ≤0.5 relative to the control group, with *p* < 0.05 according to a *t*-test [53].

## 5. Conclusions

This study used stress-tolerant LJ and stress-sensitive 218TR alfalfa plants as experimental materials to elucidate the mechanism underlying alfalfa responses to saline–alkaline stress combined with cold stress. The results not only clarified the synergistic inhibitory effect of the combined action of stress on the two alfalfa cultivars but also analyzed the mechanism of LJ’s better stress resistance compared to 218TR cultivars, and explored the key genes that may tolerate the combined stresses. Experimental analysis showed that compared with single stresses, the combined stresses caused great damage to alfalfa as shown by the phenotypic and physiological indexes. A reason that the tolerance of LJ to combined stress was stronger than that of 218TR might be that more metabolites in LJ were used to alleviate stress and reduce damage, thus improving the stress resistance of LJ. Additionally, the study data confirmed that SA is a core metabolite for alfalfa responses to multiple stresses, with its biosynthesis mainly regulated by the PAL pathway. Significant increases in *PAL1*, *PAL2*, and *ICS1* expression can enhance plant stress resistance by promoting SA synthesis. Moreover, PAL pathway-dominated SA synthesis is a key regulatory node for the stress tolerance of LJ. This research provides an important theoretical basis and core targets for breeding alfalfa varieties tolerant to both saline–alkaline stress and cold stress.

## Figures and Tables

**Table 1:** Upregulated and downregulated metabolites in LJ and 218TR under different saline–alkaline and cold stress conditions.

Control Group	Total Substance Count Detection	Number of Upregulated Substances	Names of Upregulated Substances	Number of Downregulated Substances	Names of Downregulated Substances	Metabolite Function
LJ-BS-25 °C group vs. LJ-1BS:3AS-25 °C group	49	18	GA1, GA4, IAA, IAA-Ala, IAA-Asp, IAA-Gly, IAA-Val, IAM, IAN, IBA, ILA, IP, IPA, iPR, OxIAA, SA, TAM, mTR	31	5-DS, ABA, ACC, BA9G, BAdo, BL, DHZR, DZ, GA20, GA3, GA7, H2JA, IAAcr, IAA-Glu, IAA-Phe, IAA-Trp, ICAld, JA, JA-Ile, KT, KTR, SAG, L-Trp, cZR, mT, oT, oTR, pT, pTR, tZ, tZR	ABA: Promotes dormancy, enhances stress resistance, regulates stomatal closure, inhibits growth. IAA, OxIAA: Promotes cell elongation and division, regulates plant growth and development. IAA-Ala, IAA-Phe, IAA-Val, IAA-Asp: IAA conjugate; involved in auxin storage, transport and homeostasis regulation. IAA-Trp: Precursor for auxin and other plant hormone synthesis. IAA-Gly, IAA-Glu: Involved in auxin storage, transport, and homeostasis regulation. IAM: Convertible to IAA; involved in auxin biosynthesis. IAAcr: Involved in auxin metabolism or acts as an auxin precursor/analog. IAN: Involved in the IAA biosynthetic pathway. IPA: Promotes rooting and cell elongation. IBA: Promotes rooting. ICAld: Involved in auxin metabolism or acts as an auxin precursor/analog. ILA: Involved in auxin homeostasis regulation. cZR: Involved in growth regulation. DZ, DHZR: Promotes cell division and bud differentiation. oT, oTR, mT, mTR, pT, pTR: Promotes cell division, induces bud differentiation, delays senescence. tZ, tZR: Promotes cell division, induces bud differentiation, delays senescence. TAM: Involved in tryptophan-dependent IAA pathway. L-Trp: Precursor for auxin and other plant hormone synthesis. BAdo, BA9G: Promotes cell division, induces bud differentiation. iPR, IP: Promotes cell division, regulates plant development. KT, KTR: Promotes cell division, delays senescence. ACC: Key intermediate in ethylene biosynthetic pathway. GA1, GA3, GA4, GA7, GA20: Promotes stem elongation, breaks dormancy, induces flowering and fruiting, regulates seed germination. JA: Regulates plant defense responses, promotes secondary metabolism, influences flowering and senescence. H2JA: Involved in JA signaling pathway regulation. JA-Ile: Regulates JA-mediated defense and developmental responses. SA: Regulates plant immune responses, promotes flowering, enhances stress resistance. SAG: Involved in SA storage and homeostasis regulation. 5-DS: Mediates mycorrhizal symbiosis, induces parasitic plant germination. BL: Promotes plant growth, enhances stress resistance, regulates cell elongation and division.
LJ-BS-25 °C group vs. LJ-BS-0 °C group	49	27	5-DS, ABA, BA9G, BAdo, GA1, GA3, GA4, GA7, IAA, IAA-Ala, IAA-Gly, IAA-Phe, IAA-Val, IAM, IAN, IBA, ICAld, IP, KT, KTR, OxIAA, SA, SAG, TAM, L-Trp, cZR, pTR,	22	ACC, BL, DHZR, DZ, GA20, H2JA, IAAcr, IAA-Asp, IAA-Glu, IAA-Trp, ILA, IPA, iPR, JA, JA-Ile, mT, mTR, oT, oTR, pT, tZ, tZR	
LJ-1BS:3AS-25 °C group vs. LJ-1BS:3AS-0 °C group	49	30	5-DS, ABA, BA9G, BAdo, GA1, GA20, GA3, GA4, GA7, H2JA, IAA-Ala, IAA-Glu, IAA-Gly, IAA-Phe, IAA-Trp, IAA-Val, IAM, IAN, IBA, ICAld, IP, JA, JA-Ile, KTR, SAG, SA, L-Trp, cZR, tZ, tZR	19	ACC, BL, DHZR, DZ, IAAcr, IAA, IAA-Asp, ILA, IPA, iPR, KT, OxIAA, TAM, mT, mTR, oT, oTR, pT, pTR	
LJ-BS-0 °C group vs. LJ-1BS:3AS-0 °C group	49	33	5-DS, ABA, BAdo, DHZR, GA1, GA20, GA3, GA4, GA7, H2JA, IAAcr, IAA-Ala, IAA-Asp, IAA-Glu, IAA-Gly, IAA-Phe, IAA-Trp, IAA-Val, IAM, IAN, ILA, IPA, iPR, JA, JA-Ile, SA, SAG, TAM, L-Trp, cZR, mTR, tZ, tZR	16	ACC, BA9G, BL, DZ, IAA, IBA, ICAld, IP, KT, KTR, OxIAA, mT, oT, oTR, pT, pTR	
218TR-BS-25 °C group vs. 218TR-1BS:3AS-25 °C group	49	14	GA1, GA3, GA4, IAA-Ala, IAA-Asp, IAM, ILA, IPA, iPR, OxIAA, TAM, cZR, SA, oTR	35	5-DS, ABA, ACC, BA9G, BAdo, BL, DHZR, DZ, GA20, GA7, H2JA, IAAcr, IAA, IAA-Glu, IAA-Gly, IAA-Phe, IAA-Trp, IAA-Val, IAN, IBA, ICAld, IP, JA, JA-Ile, KT, KTR, SAG, L-Trp, mT, mTR, oT, pT, pTR, tZ, tZR	
218TR-BS-25 °C group vs. 218TR-BS-0 °C group	49	20	ABA, GA1, GA3, GA4, GA7, H2JA, IAAcr, IAA-Ala, IAA-Phe, IAM, IAN, IBA, IP, JA-Ile, KTR, SA, TAM, L-Trp, cZR, pTR	29	5-DS, ACC, BA9G, BAdo, BL, DHZR, DZ, GA20, IAA, IAA-Asp, IAA-Glu, IAA-Gly, IAA-Trp, IAA-Val, ICAld, ILA, IPA, iPR, JA, KT, OxIAA, SAG, mT, mTR, oT, oTR, pT, tZ, tZR	
218TR-1BS:3AS-25 °C group vs. 218TR-1BS:3AS-0 °C group	49	36	ABA, ACC, BA9G, BAdo, DZ, GA3, GA4, GA7, H2JA, IAAcr, IAA-Asp, IAA-Glu, IAA-Gly, IAA-Phe, IAA-Trp, IAM, IAN, IBA, ICAld, IP, iPR, JA, JA-Ile, KT, SA, SAG, TAM, L-Trp, cZR, mT, mTR, oT, oTR, pT, pTR, tZR	13	5-DS, BL, DHZR, GA1, GA20, IAA, IAA-Ala, IAA-Val, ILA, IPA, KTR, OxIAA, tZ	
218TR-BS-0 °C group vs. 218TR-1BS:3AS-0 °C group	49	34	ABA, BA9G, DHZR, GA3, GA4, GA7, H2JA, IAA-Ala, IAA-Asp, IAA-Glu, IAA-Gly, IAA-Phe, IAA-Trp, IAM, IAN, IBA, ICAld, ILA, IPA, iPR, JA, KT, OxIAA, SAG, TAM, L-Trp, cZR, mT, mTR, SA, oT, oTR, pT, pTR	15	5-DS, ACC, BAdo, BL, DZ, GA1, GA20, IAAcr, IAA, IAA-Val, IP, JA-Ile, KTR, tZ, tZR	

**Note:** ABA for Abscisic acid; IAA for Indole-3-acetic acid; OxIAA for 2-Oxindole-3-acetic acid; IAA-Ala for N-(3-Indolylacetyl)-L-alanine; IAA-Phe for N-(3-Indolylacetyl)-L-phenylalanine; IAA-Val for N-(3-Indolylacetyl)-L-valine; IAA-Asp for Indole-3-acetyl-L-aspartic acid; IAA-Trp for Indole-3-acetyl-L-Tryptophan; IAA-Gly for Indole-3-acetyl glycine; IAA-Glu for Indoleacetyl glutamic acid; IAM for 3-Indoleacetamide; IAAcr for 3-Indoleacrylic acid; IAN for 3-Indoleacetonitrile; IPA for 3-Indolepropionic acid; IBA for Indole-3-butyric acid; ICAld for Indole-3-carboxaldehyde; ILA for Indole-3-lactic acid; cZR for cis-Zeatin riboside; DZ for Dihydrozeatin; DHZR for Dihydrozeatin ribonucleoside; oT for ortho-Topolin; oTR for ortho-Topolin riboside; mT for meta-Topolin; mTR for meta-Topolin riboside; pT for para-Topolin; pTR for para-Topolin riboside; tZ for trans-Zeatin; tZR for trans-Zeatin riboside; TAM for Tryptamine; L-Trp for L-Tryptophan; BAdo for 6-Benzyladenosine; BA9G for N6-Benzyladenine -9-Glucoside; iPR for Isopentenyladenosine; IP for N6-Isopentenyladenine; KT for Kinetin; KTR for Kinetin riboside; ACC for 1-Aminocyclopropanecarboxylic acid; GA1 for Gibberellin A1; GA3 for Gibberellin A3; GA4 for Gibberellin A4; GA7 for Gibberellin A7; GA20 for Gibberellin A20; JA for Jasmonic acid; H2JA for Dihydrojasmonic acid; JA-Ile for Jasmonoyl-L-Isoleucine; SA for Salicylic acid; SAG for Salicylic acid 2-O-β-Glucoside; 5-DS for 5-Deoxystrigol; BL for Brassinolide.

## Data Availability

The original contributions presented in this study are included in the article. Further inquiries can be directed to the corresponding author(s).
